# Synaptophysin is involved in resetting of the mammalian circadian clock

**DOI:** 10.1186/1740-3391-11-11

**Published:** 2013-10-01

**Authors:** Marie Aramendy, Sascha Seibert, Philipp Treppmann, Karin Richter, Gudrun Ahnert-Hilger, Urs Albrecht

**Affiliations:** 1 Department of Biology, Unit of Biochemistry, University of Fribourg, Fribourg, Switzerland; 2 AG Functional Cell Biology, Institute for Integrative Neuroanatomy, Charité Centre for Basic Medicine, Berlin, Germany

**Keywords:** Light, Synaptic vesicles, Synaptobrevin

## Abstract

**Background:**

Mammals can adapt to changing light/dark conditions by advancing or delaying their circadian clock phase. Light pulses evoke changes in gene expression and neuronal activity in the suprachiasmatic nuclei (SCN), the central pacemaker of the circadian system. Alterations in neuronal activity are partially mediated by changes in synaptic vesicle (SV) fusion at the presynaptic membrane, which modulates release of neurotransmitters.

**Methods:**

Male synaptophysin (Syp) knock-out and littermate control wild type mice were tested in an Aschoff type I resetting paradigm. Additionally, gene expression of *cFos*, *Per1* and *Per2* was assessed in the SCN. Finally, complexes between the synaptic vesicle proteins Syp and synaptobrevin (Syb) were studied in order to correlate behavior with protein complexes at synaptic vesicles.

**Results:**

Here we show that mice lacking Syp, a modulator of neurotransmitter release, are defective in delaying clock phase. In contrast, clock phase advances as well as clock period are normal in *Syp*^*-/-*^ knock-out mice. This correlates with the formation of Syp/Syb complexes.

**Conclusions:**

Our findings suggest that Syp is involved specifically in the response to a nocturnal light pulse occurring in the early night. It appears that the SV component Syp is critically involved in the delay portion of the resetting mechanism of the circadian clock.

## Background

Circadian pacemaking in the suprachiasmatic nucleus (SCN), the main coordinator of circadian rhythms in mammals, arises from cellular transcriptional/posttranslational autoregulatory feedback loops [1]. These rhythms in SCN neurons can be aligned to the environmental light/dark cycle. Light signals are transmitted from the retina via the retinohypothalamic tract to a subset of SCN neurons leading to the activation of several signaling pathways that evoke chromatin remodeling and the induction of immediate early genes and clock genes [2]. Spreading of this information from light receptive SCN neurons to their neighbors eventually evokes a change of clock phase in the SCN, ultimately leading to a behavioral change that can be readily observed as a change in onset of wheel running activity [3].

Circadian communication between SCN neurons and their targets relies upon daily cycles of electrical firing and secretion of neurotransmitters [4]. How the molecular clock drives secretion of neurotransmitters is not clear, although a number of gene and protein profiling studies identified several genes [5] and proteins [6] in the SCN to be expressed in a time of day dependent manner. Among these genes and proteins, factors involved in the function of synaptic vesicles (SVs) are of particular interest, because SVs are responsible for the transmission of information from one cell to another in the brain. In neurons, information arrives at the presynaptic terminal in the form of an action potential and is transmitted to the postsynaptic cell via neurotransmitters. In the presynaptic terminal, these neurotransmitters are packaged into SVs. The action potential triggers SV exocytosis into the synaptic cleft, where the released neurotransmitters activate postsynaptic receptors and elicit a cellular response [7]. In this process, the regulation of the filling state and fusion of SVs with the plasma membrane and their endocytic retrieval are critical. The filling state of SVs appeared to be regulated by light involving the clock gene *Per2*[8]. The complete endocytic retrieval of SV components was observed to be in a time-of-day dependent manner. In this context, not all SV proteins appeared to obey the same time course of endocytic retrieval, as evidenced by a specific sorting of the vesicular glutamate transporter to the plasma membrane [9]. Furthermore, proteomic analysis revealed a role of SV cycling in sustaining the SCN circadian clock [10].

In this study, we investigated the role of synaptophysin (Syp) and its potential role in the circadian clock. Syp is a permanent molecular component present selectively on SVs and therefore is used as one of the main SV markers. Indeed it is the most abundant SV protein with respect to molecular mass [11]. Several lines of evidence implicate Syp in the regulation of neurotransmitter release. Syp appeared to regulate Ca^2+^ induced neurotransmitter release [12,13] and it seemed to be required for doing this in an exocytotic manner [14]. In addition, Syp modulates the formation of synaptic contacts [15] and is specifically involved in the endocytic retrieval of synaptobrevin [16]. Furthermore, Syp regulates the kinetics of synaptic vesicle endocytosis in neurons [17]. Overexpression of Syp resulted in an increase of amplitude and reduced delay of onset of evoked synaptic currents in spinal motor neurons [18]. However, deletion of Syp in mice did not affect SV formation and function in these animals [19,20] probably due to redundancy with other SV molecules [21]. Overall, these results indicate that Syp may be involved in the coupling of excitation and secretion, probably by controlling either fusion probability or endocytic retrieval of SV proteins, thereby modulating the supply of SVs primed for release. In addition, SV of rod photoreceptors are morphologically altered in Syp-deletion mutants which may have some impact to light perception and behavior in the context of a day night cycle [22].

Because resetting of the circadian clock in response to a light pulse requires rapid transmission of electrical signals via synaptic release of neurotransmitters [23], we investigated whether Syp may play a role in this process.

## Methods

### Animals

All animal work was performed in accordance with the guidelines of the Schweizer Tierschutzgesetz (TSchG, SR455, Abschnitt 2: Art. 5 + 7, Abschnitt 5: Art. 11 and Abschnitt 6: Art.12-19) and was approved by the state veterinarian of Fribourg (permit FR 101/10) and in accordance with the declaration of Helsinki. Only male mice were used in this study. Animals were raised in LD 12:12h and were not in a different cycle before the DD experiments.

The *Syp*^*-/-*^ knock-out mice [20] used in this study were bread from homozygous animals to obtain wild-type littermates with a matching genetic background (C57Bl6/J). The genotype of the offspring was determined by PCR. The PCR protocol for *Syp* was according to [24]. The following primers were used:

*Syp*_1: 5′-GCA TGA CCC CTC TGT CTG AT-3′

*Syp*_2: 5′-TGA ATG AAC TGC AGG ACG AG-3′

The final primer concentration was 1.0 μM*.* The dNTP (Roche) concentration was 0.4 mM*.* The final MgCl_2_ concentration was 3.0 mM. To improve annealing, 6 nM (NH_4_)_2_SO_4_ was added to the PCR reaction mix*.* 2.5 U *Taq* DNA polymerase (Qiagen) were used per 50 μl reaction. A final concentration of 0.25x and 0.2x Q-solution (Qiagen) was used to increase PCR specificity. An initial denaturation was done at 94°C for 2 min. Subsequent denaturation was done at 94°C for 30 s followed by an annealing step of 30 s. The annealing temperature was 56°C for *Syp.* The elongation step was performed at 72°C for 1 min. After 34 cycles, the PCR was ended with a final extension at 72°C for 10 min.

The *Per2* mutant mice used in this study are described in [25].

### Subcellular fractionation

SV were prepared at 4° from adult mouse whole brains in the presence of protease inhibitors following the procedure described [15]. Mice were sacrificed at the given time points in the light/dark cycle (Zeitgeber time, ZT). The obtained SV fractions were immediately subjected to cross-linking using disuccinimidyl suberate (DSS) as described earlier [15]. Generally, wild-type and *Per2* mutant mice were analyzed in parallel [8]. Protein determination was performed from the individual membrane fractions and equal amounts of protein were loaded for SDS-PAGE. For each set of experiments, membrane fractions were run in parallel, and determination of SNAP25 (synaptosomal-associated protein 25) was used as an internal reference.

### Locomotor activity monitoring and circadian phenotype analysis

Mice housing and handling were performed as described earlier [26]. Animals were entrained in LD 12:12h for 7–15 days before they were released into constant darkness (DD). Activity was assessed with a running-wheel and evaluated using the ClockLab software package (Actimetrics). Activity records were double plotted in threshold format for 6-min bins. Period length was assessed by χ^2^ periodogram analysis for days 4–10 in DD. To determine light induced phase shifts (white light, 500 lux [27]), an Aschoff Type I protocol was used [26]. Animals were allowed to stabilize their free-running rhythm for at least 1 month prior to the light pulse. The circadian time (CT) at the beginning of the light pulse was calculated for every mouse individually. The phase response curve was established administering 15 min light pulses at CT10 (N [wild-type/*Syp*^*-/-*^] = 11/7), CT14 (N = 12/8), and CT22 (N = 12/7).

### *In situ* hybridization

Locomotor activity was monitored for each mouse to properly determine activity onsets, which is necessary to calculate CT values. For light induction experiments, animals were kept in DD for about 1 month before they were exposed to a 15 min light pulse (400 lux) at different CTs. 45 min after the end of the light pulse, the mice were first anesthetized with Attane™ Isoflurane (Provet AG) and then sacrificed. Control animals were sacrificed without prior light exposure.

Specimen preparation and *in situ* hybridization were carried out as described previously [28]. Briefly, the ^35^S-UTP (1250 Ci/mmol, PerkinElmer) labelled riboprobes were synthesized using the RNAMaxx™ High Yield transcription kit (Stratagene) according to manufacturer’s protocol. The *Per1* probe was made from a cDNA corresponding to nucleotides (nt) 620–1164 (accession no. AF022992), *Per2* to nt 229–768 (AF036893), *cFos* to nt 237–332. 7 μm thick paraffin sections were dewaxed, rehydrated and fixed in 4% paraformaldehyde. Sections were then permeabilized using a proteinase K (Roche) digestion (20 μg/ml in 50 mM Tris/HCl 5 mM EDTA pH8, for 5 min) before they were fixed again and acetylated. After serial dehydration, hybridization was performed over-night at 55°C in a humid chamber. Stringency washes were carried out at 63°C. Slides were subjected to a ribonuclease A (Sigma) digestion and then dehydrated in graded ethanol series. Quantification was performed by densitometric analysis (GS-800, BioRad) of autoradiography films (Amersham Hyperfilm) using the Quantity One software (BioRad). Data from the SCN were normalized subtracting the optical density measured in the lateral hypothalamus next to the SCN. For each experiment, at least 3 animals per genotype were used and 4 to 9 adjacent SCN sections per animal were analyzed. Relative RNA abundance values were calculated by defining the highest wild-type mean value of each experiment as 100%. For statistical analysis, all normalized values obtained for one brain were averaged to obtain one final value per animal.

### Transactivation assays

A 1.4 kb fragment of the mouse *Syp* promoter region was cloned into the pGL3 basic vector (Promega) containing the firefly luciferase reporter gene. Full-length mouse cDNAs encoding *mBmal1* (NM_007489), *mNpas2* (BC109166) and bacterial beta-galactosidase were cloned into the pSCT1 vector. Transfection experiments were performed as described previously [29].

### Statistical analysis

Significant differences were determined using GraphPad Prism 4 software. Depending on the type of data, either unpaired t-test, one- or two-way ANOVA with Bonferroni post-test was performed. Values were considered significantly different with p < 0.05 (*), p < 0.01 (**), or p < 0.001 (***).

## Results

**Figure 1:**
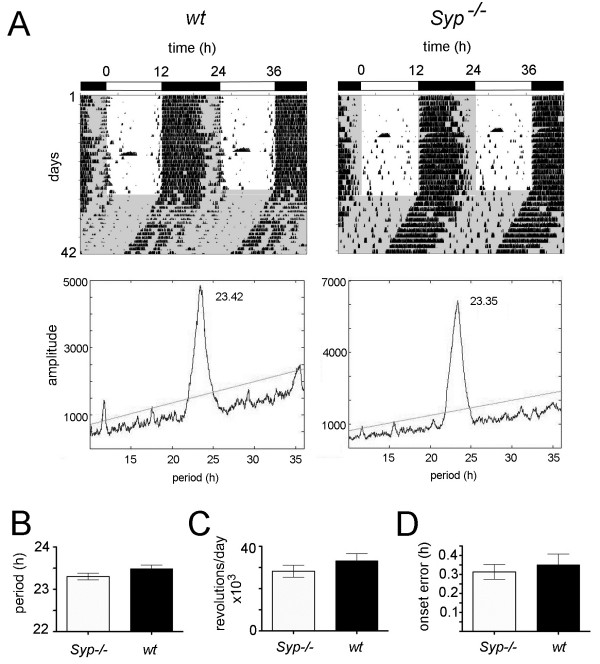
**Locomotor activity and clock period. (A)** Representative locomotor activity records of wild-type (wt) (left panel) and synaptophysin knock-out (*Syp*^*-/-*^) male mice (right panel). Black and white bars on top of the plots indicate the 12 h light: 12 h dark cycle (LD). The grey shaded areas filling the panels in the lower part indicate constant darkness (DD). Below the locomotot activity records χ^2^-periodogram analysis is shown indicating the period length. **(B)** Period is similar in wild-type (n=17) and *Syp*^*-/-*^ male mice (n=18). **(C)** Total activity is comparable between the two genotypes. **(D)** Precision of activity onset is comparable between the two genotypes.

To assess the importance of Syp on circadian behavior, we compared *Syp*^*-/-*^ knock-out mice with corresponding wild-type littermates. In a first step, we characterized clock parameters including period, total daily wheel-running activity and precision of daily onset of activity. The period of *Syp*^*-/-*^ knock-out mice was 23.3 ± 0.08 h, and of wild-type animals was 23.49 ± 0.09 h. An unpaired t-test with n = 18 *Syp*^*-/-*^ and 17 male wild-type animals revealed no difference in period between the two genotypes (p > 0.05) (Figure 1A,B). Also total activity assessed by wheel-revolutions was not different between the two genotypes: *Syp*^*-/-*^ = 28’241 ± 2850 revolutions/day and wild-type = 33’175 ± 3392 revolutions/day, p > 0.05 (unpaired t-test), n = 18 *Syp*^*-/-*^ and 17 wild-type mice (Figure 1C). Precision of activity onset was also measured at the subjective day/night transition (CT12). This parameter was not different between *Syp*^*-/-*^ knock out (0.31 ± 0.04 h) and wild-type mice (0.35 ± 0.06 h), as revealed by an unpaired t-test, p > 0.05, n = 18 *Syp*^*-/-*^ and 17 wild-type mice (Figure 1D). Taken together, we find that under non-challenging conditions *Syp*^*-/-*^ knock-out mice behave normally.

**Figure 2:**
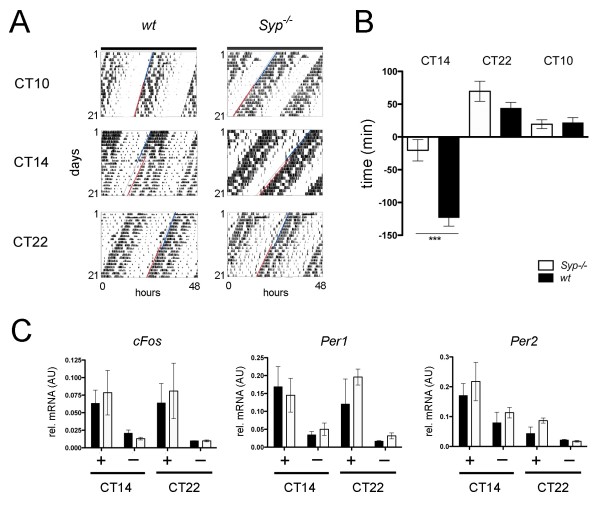
**Phase shifting response to light pulses and mRNA accumulation in the SCN. (A)** Representative actograms of wild-type (wt) and *Syp*^*-/-*^ knock-out mice with light pulses at CT10 (top panels), CT14 (middle panels) and CT22 (bottom panels). Blue lines trace activity onset before the light pulse and red lines trace activity onset after the light pulse. **(B)** Quantification of the phase shifts in *Syp*^*-/-*^ knock-out (white bar) and wild-type mice (black bar). *Syp*^*-/-*^ knock-out mice show a significantly reduced phase delay in response to light at CT14 (*Syp*^*-/-*^ = -34 ± 12 min.; wt = -122 ± 10 min., unpaired t-test p < 0.001 = ***, n = 8 *Syp*^*-/-*^ and 12 *wt*). No difference between the genotypes was observed at CT22 (*Syp*^*-/-*^ = 60 ± 12 min.; wt = 45 ± 6 min., n = 7 *Syp*^*-/-*^ and 12 *wt*) and CT10 (*Syp*^*-/-*^ = 26 ± 12 min.; wt = 21 ± 8 min., n = 7 *Syp*^*-/-*^ and 11 *wt*). **(C)** mRNA accumulation in the SCN of *Syp*^*-/-*^ knock-out (white bar) and wild-type mice (black bar) before (-) and after (+) a 15 minute light pulse at CT14 or CT22. No differences in expression of *cFos*, *Per1* or *Per2* can be observed (n = 3, two-way ANOVA with Bonferroni post-test). Mean values are represented as SEM.

Next, we investigated a potential role of Syp under challenging conditions. Light induced resetting of circadian behavior represents such a challenge. The animals were exposed to light and, as a consequence, adjusted their clocks according to the time when the pulse of light occurred. If the light pulse was given at the subjective day that is between circadian time (CT) 0 and CT12, the animals do not respond to light, as evidenced by a light pulse at CT10 given to wild type and *Syp*^*-/-*^ knock-out mice (Figure 2A,B). Application of a 15 minute light pulse in the early subjective night at CT14 evoked in both genotypes as expected a phase delay. This phase delay, however, was significantly reduced in *Syp*^*-/-*^ knock-out animals (p<0.001) (Figure 2A,B). In contrast, a light pulse in the late subjective night at CT22 advanced activity onset with no significant difference between wild-type and *Syp*^*-/-*^ knock-out mice (p>0.05) (Figure 2A,B). Overall, we observed a defect in clock resetting in *Syp*^*-/-*^ knock-out mice at CT14 but not at CT22.

In order to test whether expression of light inducible genes is altered in the SCN of wild-type and *Syp*^*-/-*^ knock-out mice, we measured mRNA levels of *cFos*, *Per1* and *Per2* genes after a nocturnal light pulse [30-32]. We found that the light induced expression of *cFos*, *Per1* and *Per2* in the SCN was similar in both genotypes (Figure 2C). These results indicate that lack of Syp does not affect light induction of these genes.

**Figure 3:**
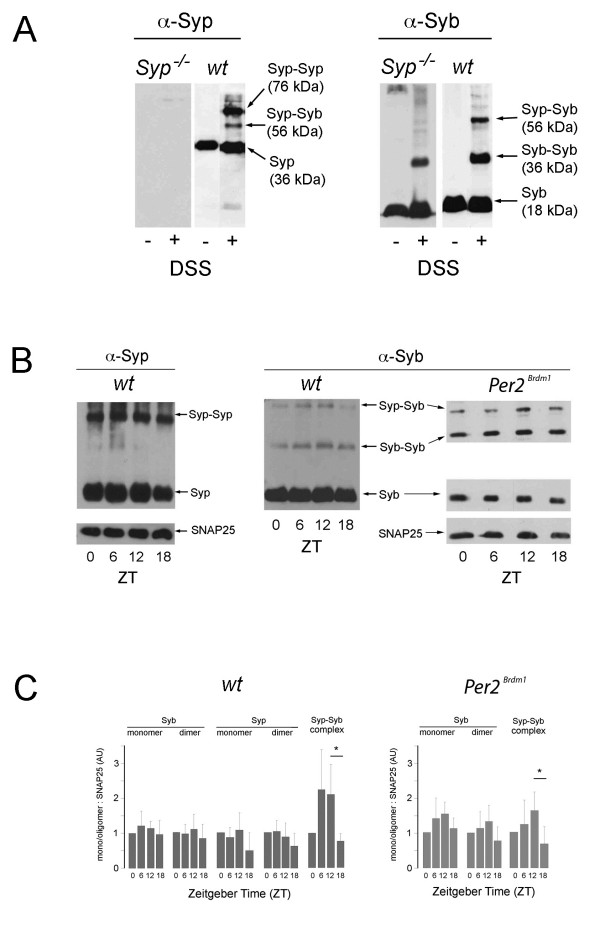
**Diurnal changes of the interaction between Synaptophysin (Syp) and Synaptobrevin (Syb). (A)** Western blots of proteins from synaptic vesicles prepared from either synaptophysin knock-out animals (*Syp*^*-/-*^), wild-type (*wt*) littermates or *Per2* mutant mice. Antibodies against synaptophysin (α-Syp, left panel) and synaptobrevin (α-Syb, right panel) reveal the presence of synaptophysin (Syp) or synaptbrevin (Syb) with (+) or without (-) chemical cross-linking using disuccimidyl suberate (DSS). Cross-linked samples show in addition to the respective monomers the Syp/Syb complex at 56 kDa and the Syp or Syb dimers. In synaptic vesicles of *Syp*^*-/-*^ animals, only Syb and its dimer can be observed. **(B)** Western blots after cross-linking of proteins show the Syp/Syb interaction over a 12 hour light/12 hour dark cycle. Synaptic vesicles were prepared at the indicated Zeitgeber times (ZT). Antibodies against Syp (left panel) and Syb (right panels) show changes in the Syp/Syb complex over time. SNAP25 served as internal control. **(C)** Quantification of Syp/Syb complexes using α-Syb antibodies. Data from three independent sets of animals are shown with each sample analyzed at least twice. SNAP25 served as reference for quantification. For each set, values were normalized to the value at ZT0 which was set to 1. While the Syp and Syb monomers and dimers do not change over time, the formation of the Syp/Syb complex appears to be time dependent with maximal values observed during the light period corresponding to the resting phase of mice (ZT6 and ZT12). Values represent the mean of three animals ± S.D. Significance was determined by student’s t-test between ZT6 and ZT18 ( p = 0.06) and ZT12 and ZT18 (p = 0.03).

**Figure 4:**
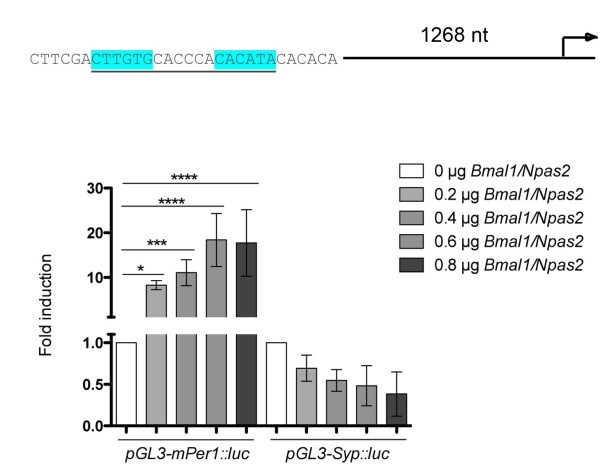
**Transactivation assay of the *Syp *promoter in NG108-15 neuroblastoma cells.** 0.5 μg of a luciferase reporter construct containing a 1.4 kb Syp promoter sequence was co-transfected with 0.2 μg, 0.4 μg, 0.6 μg and 0.8 μg of *Bmal1* and *Npas2* expression constructs, respectively. As control 0.5 μg the mouse *Per1* luciferase reporter construct was used. Duplicates are represented as mean ± SD. One-way ANOVA was performed for statistical analysis. *p ≤ 0.05; **p ≤ 0.01; ***p ≤ 0.001; ****p ≤ 0.0001.

Syp modulates the fusion of SVs with the membrane of neurons (reviewed in [21]) partially via a heterodimeric complex with synaptobrevin (Syb, VAMP2) [15]. In order to correlate the formation of hetero- and homodimeric complexes of Syp with the resetting phenotype observed in Figure 2, we analyzed Syp complex formation in wild-type and *Per2* mutant mice, which show a similar resetting phenotype as *Syp*^*-/-*^ knock-out mice [33]. As expected, Syp interacts with itself and with Syb in wild-type mouse brains (Figure 3A). However, the formation of the Syp/Syb heterodimer but not the Syp/Syp and Syb/Syb homodimer is time of day dependent (p<0.05) (Figure 3B,C, left panels). Interestingly, the amount of Syp/Syb heterodimers appears to be reduced but still time of day dependent in mice mutant in the clock component *Per2* (Figure 3B,C, right panels), indicating that *Per2* may affect in an unknown manner the formation of Syp/Syb heterodimers. Mutation of *Per2* does not affect the amounts of Syp and Syb proteins (Figure 3B,C) and in transactivation assays the *Syp* promoter was not regulated by clock components (Figure 4). These results indicate that *Per2* may influence Syp/Syb heterodimer formation at the posttranslational level. Taken together, we find that Syp interacts with Syb in a time of day dependent fashion and that this interaction is modulated by an unknown mechanism involving *Per2*. This correlates with the behavioral resetting phenotype of *Syp*^*-/-*^ knock-out and *Per2* mutant animals, which both show reduced phase delays in response to early nocturnal light. However, we do not understand how Syp/Syb complex formation and resetting are mechanistically linked.

## Discussion

Despite its broad abundance, a physiological function of Syp is lacking so far. There is only one report on behavioral changes showing that Syp deletion mutants are more explorative but have deficits in some learning paradigms [34]. We report here for the first time a role of *Syp* in clock resetting. Although the circadian clock appears to be normal in these mice (Figure 1), the clock resetting mechanism in response to a nocturnal light pulse in the early subjective night is abnormal (Figure 2A,B). The *Syp*^*-/-*^ knock-out mice show a reduced phase delay response at the behavioral level. Interestingly, genes inducible by a nocturnal light pulse in the SCN such as *cFos*, *Per1* and *Per2* are normally induced (Figure 2C) in a manner comparable to previous studies [31,32,35,36]. Because synchronization between neurons in the SCN involves synaptic signaling (reviewed in [4]), it is likely that Syp plays a role in the orchestration for spreading the light induced signal within the SCN and other brain regions involved in the regulation of locomotor activity. This view is supported by our observation that the complex formed between Syp and Syb is time of day dependent (Figure 3). Interestingly, the protein levels of both Syp and Syb are not variable over time, indicating that it is the formation of the complex between the two proteins that may be responsible for coordinating the behavioral response to light. The destruction of the complex is critical in that sense, that neurotransmitter release involves separation of the Syp-Syb complex. Syb can then interact with the SNARE complex to promote vesicle fusion. However, it is not known how this process is temporally regulated. One hypothesis is that the circadian clock may affect the formation of this complex. This is supported by the observation that the Syp-Syb interaction is high during the day when animals are in their resting phase. The slightly lower amounts of Syp-Syb complex observed in *Per2* mutant mice would be indicative of such a scenario (Figure 3C). However, further experiments are needed to unravel the mechanistic relationship between clock resetting and Syp function.

In summary we show here that deletion of Syp in mice affects resetting of the circadian clock in response to a light pulse during the early subjective night. This may be related to an alteration in the formation of Syp-Syb heterodimers, which are hypothesized to be involved in the fusion of SVs to the plasma membrane and/or recovery of SVs from the plasma membrane [21].

## Competing interests

The authors declare that they have no competing interests.

## Authors’ contributions

GA and UA planned and guided the study, analyzed the data with MA and SS and wrote the manuscript. MA, SS, PT and KR performed the experiments. All authors read and approved the manuscript.
